# Up–Down Reader: An Open Source Program for Efficiently Processing 50% von Frey Thresholds

**DOI:** 10.3389/fphar.2018.00433

**Published:** 2018-05-01

**Authors:** Rafael Gonzalez-Cano, Bruno Boivin, Daniel Bullock, Laura Cornelissen, Nick Andrews, Michael Costigan

**Affiliations:** ^1^Kirby Neurobiology Center, Boston Children’s Hospital and Department of Neurobiology, Harvard Medical School, Boston, MA, United States; ^2^Department of Anesthesia, Boston Children’s Hospital, Harvard Medical School, Boston, MA, United States

**Keywords:** up–down, von Frey, free software, behavior, tactile, mechanical, allodynia, QST

## Abstract

Most pathological pain conditions in patients and rodent pain models result in marked alterations in mechanosensation and the gold standard way to measure this is by use of von Frey fibers. These graded monofilaments are used to gauge the level of stimulus-evoked sensitivity present in the affected dermal region. One of the most popular methods used to determine von Frey thresholds is the up–down testing paradigm introduced by Dixon for patients in 1980 and by Chapman and colleagues for rodents in 1994. Although the up–down method is very accurate, leading to its widespread use, defining the 50% threshold from primary data is complex and requires a relatively time-consuming analysis step. We developed a computer program, the Up–Down Reader (UDReader), that can locate and recognize handwritten von Frey assessments from a scanned PDF document and translate these measurements into 50% pain thresholds. Automating the process of obtaining the 50% threshold values negates the need for reference tables or Microsoft Excel formulae and eliminates the chance of a manual calculation error. Our simple and straightforward method is designed to save research time while improving data collection accuracy and is freely available at https://sourceforge.net/projects/updownreader/ or in supplementary files attached to this manuscript.

## Introduction

Touch is one of our most utilized senses, often so ubiquitous that we forget that we continuously process information this way while conscious. Given our inevitable physical contact with the environment, marked alterations in this sensory parameter can be extremely disabling to those who suffer these symptoms. Tactile hyper- or hypo- sensitivity can be the result of rare congenic conditions (Bennett and Woods, 2014) or more commonly through disease, both inflammatory (Woolf and Salter, 2000) and neuropathic (Costigan et al., 2009). Furthermore, the two most disabling symptoms of neuropathic pain experienced by patients are tactile hypersensitivity and spontaneous pain (Loach et al., 2001). Mechanosensitive alterations are also key to the sensory abnormalities produced by most pathological pain models in rodents (Mogil, 2009).

Cutaneous sensitivity testing with von Frey filaments provides a quantitative measurement of sensory threshold. They are readily used to evaluate physiology underlying sensory abnormalities, ranging from numbness to hyperalgesia or allodynia (Maag and Baron, 2006; González-Cano et al., 2013; Hockley et al., 2017). As such, they have become a mainstay of clinical and pre-clinical research of mechanosensation (Le Bars et al., 2001; Cornelissen et al., 2013, 2014). Clinical studies of cutaneous sensitivity rely on self-report, i.e., presence or absence of a response to a stimulus. In rodent pain models, tactile sensitivity is usually expressed as the paw withdrawal threshold of the experimentally manipulated hind limb. Different methods can be used to estimate such thresholds; however, the up–down method first introduced by Dixon (1980) and subsequently modified by Chaplan for use in rodents (Chaplan et al., 1994) remains among the most commonly used. Indeed, a recent survey indicated that approximately 60% of publications where paw withdrawal threshold was measured used the up–down method or a modified version of it (Mills et al., 2012).

To streamline the calculation of the mechanical sensory thresholds following data acquisition, we have developed a computer program that can locate and recognize handwritten von Frey assessments from a scanned PDF document and translate these measurements into 50% pain thresholds based on the up–down method. The input grid is first parsed into individual cells utilizing the commonly used computer vision library, OpenCV 3.1^1^. The content of the cells is then identified using pre-trained machine learning algorithms to recognize X and O values entered into the table by the investigator. The Up–Down Reader (UDReader) then automatically calculates and reports the 50% thresholds, reducing processing time and decreasing errors produced during the data analysis process.

## Description of the System

The program was developed in Python 2.7^2^ and released under MIT license. Versions compatible with most common operating systems (MacOS and Windows) are available. The program is able to evaluate 50% von Frey thresholds in three species (mice, rats, or humans) depending on the scoring template used. The evaluation of mechanical sensitivity via von Frey filaments must be recorded on templates designed for this application, which are available within the program in PDF format and printed on paper for use. Tables within these sheets allow forces ranging from 0.04 to 4 g (0.39–39.2 mN) to be recorded in mice and forces of 0.6 to 15 g (5.88–147 mN) to be documented in rats. Tables also allow recording of human mechanical detection threshold (MDT) using forces from 0.02 to 1.4 g (0.20–13.7 mN) as well as mechanical pain threshold (MPT) using forces from 4.0 to 180 g (39.2–1,766 mN). The software works with all commercially available von Frey hairs provided that the researcher applies the forces indicated in the testing sheets. The processing portion of UDReader is independent of the set of filaments used.

**FIGURE 1:**
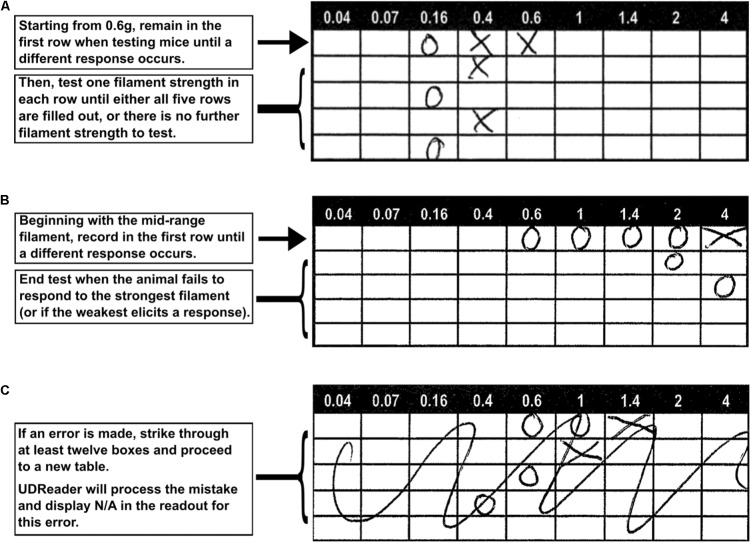
Instructions for using the supplied template sheets. In mice, the 0.6 g von Frey filament is tested first, and the result is recorded in the first row. Further tests are also recorded in this row, up until and including the first test that evokes a different response in the mouse. Results from the next four filament tests are then recorded in the following four rows to complete the trial for an animal **(A)**. If the subject’s sensitivity range exceeds that of the von Frey filaments, then this is recorded in the table and the testing concludes. The remaining rows are not filled **(B)**. If a mistake is made while recording, the table should be crossed out. UDReader will process the mistake and display “N/A” when over 12 boxes are struck through **(C)**.

Calibrated von Frey filaments must be applied as previously described by Chaplan et al. (1994). Briefly, testing is initiated using the filament force in the mid-range [mouse: 0.6 g (5.88 mN); rats: 4 g (39.23 mN); and humans: MDT: 0.4 g (39.2 mN), or MPT: 8 g (78.5 mN)]. The fiber is gently pushed against the surface of the skin from below. A positive response in the animal model is a flinch of the leg indicating that it has clearly perceived the stimulus; a verbal “Yes” expresses a positive response from a human participant. The number of individual stimulations and the time between each von Frey filament application varies and is determined by the experimental operator. Some determine a positive response as greater than 50% positive limb movements in response to 10 individual filament applications, others test as low as a single application per filament (Higgins et al., 2015; Xie et al., 2015; Li et al., 2016; Mangione et al., 2016). Once the reaction to that filament is determined, the response is recorded as either an X for a positive response or an O for a negative response. The method of recording positive and negative responses is shown in **Figure 1** and described below. As UDReader recognizes strings of X’s and O’s produced in each table from a dictionary of all possible correctly tabulated responses, it is important that the investigator does not deviate from the approach given.

A potential limitation of the software is that UDReader requires the forces tested to be the same as those indicated in the pre-determined sheet. For this reason, we included different sheets designed for mice, rats, and humans which center on the most common range of forces used in these species. However, it is not yet possible to alter these parameters if these differ from forces used in a study. We are considering a function to do so in a future version of the program.

### Data Collection

Filling out a complete table (**Figure 1A**):

(1) As an example, in mice, the first test will be the midrange 0.6 g filament. If this elicits a response, then an X is placed in the 0.6 g box in the first row.
(2) In this case, the next weaker filament (0.4 g) is tested. If the mouse responds, an X is placed in the first row 0.4 g box.
(3) In the following test, if there is no response to the next weaker filament, an O is placed in the first row and the next stronger fiber is tested.
(4) Depending on the mouse’s response, either an O or X is recorded in the second row. The next stronger filament is then tested if there is no response, or a weaker filament is used if a response occurs. The result is recorded in row three.
(5) This process is repeated for two more rounds and the results are recorded in row four and five, at which stage the test is completed.

Completing a table before reaching the final row (**Figure 1B**):

(1) In the case of an insensitive mouse, the first row will contain negative responses, marked by O’s moving successively right until one filament elicits a response and is marked with an X. Then, the next weaker fiber is tested, and the response is marked in row two.
(2) If the mouse does not respond to the strongest filament, the testing concludes, regardless of which row this result is achieved in.

Correctly resolving a mistake in a table (**Figure 1C**):

(1) The best way to correct a recording error is to draw a large cross (or other graphic) over the erroneous table so that over 12 individual boxes are marked. If this occurs, the program will read the table result as an error and replace the 50% value with N/A (for Not Applicable) in the results readout.
(2) Following this, move to the next table and re-perform the analysis, marking the correct boxes.

#### Data Upload

After the evaluation of one or more subjects (for animal models, one table per animal, 20 individual tables per template sheet; for human participants, 1 row of 2 tables per human, 10 subjects per template sheet), the sheets are scanned as multipage or single page PDF documents. Once scanned, this file can easily be loaded into the UDReader program, which will automatically calculate each table’s 50% threshold.

#### Running the UDReader Software

From a simple and uncluttered interface, the program first requests a path to an input file and then processes all of the pages contained in the document. Machine learning k-NN (nearest neighbor) algorithms are employed by the UDReader program to recognize and classify handwritten characters as X’s or O’s. An ensemble method subsequently aggregates the results, increasing character identification performance. The k-NN algorithm was used over other classifiers for its simple yet powerful classification method on a small number of classes, leading to a robust symbol classification with minimal error. The program converts each page within the PDF file to a wraped image, extracting and retaining only the grid of interest, and then parses cells identified in each experiment table. The contents of each cell within a table are classified as X, O, or empty, and the program groups the values within each defined table as string sequences. In the event where the strings of X’s and O’s obtained are not present in the pre-computed list of valid sequences, the Levenshtein algorithm is used to measure their similarity to the predefined valid sequences, and then rectifies the deviations based on a closest-match approach. This step helps address potential character recognition failures and reduce sequence misidentifications. The final sequence is tabulated in a dictionary which applies the Dixon formula:

$$50\%\;threshold\;(g)=10^{X_{f}+\kappa \delta }/10,000$$

where *X*_f_ = value (in log units) of the final von Frey filament used; κ = tabular value (for the pattern of positive/negative responses); and δ = mean difference (in log units) between stimuli (here, 0.25 for mice, 0.17 for rats and 0.25 and 0.21 for human MDT and MPT respectively). Individual values are given for each table processed, until the full sheet of tables is processed. This process is repeated for each page of the input document.

#### Data Output

Results are presented on the GUI in a scrollable pane. UDReader includes the option to save the results to a CSV file in the same location as the PDF.

## Testing the System

**FIGURE 2:**
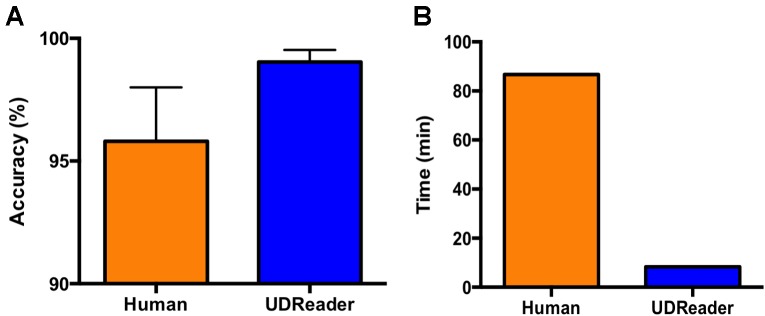
UDReader produces faster and more accurate results than a human tester. Comparison of the accuracy **(A)** and the time expended **(B)** by human evaluation and UDReader in processing 41 sheets containing 672 table results. Mice evaluated were both SNI and sham animals, and von Frey filaments were applied to the left hind paw. Results demonstrate the mean accuracy per sheet. Error bars represent mean ± SEM.

To test the accuracy of UDReader, we used identical data sheets to record results from mice with SNI (spared nerve injury) and sham animals tested in the left hind paw. After analyzing the von Frey values obtained from human testers using the traditional approach, we compared these results with those given by the application. The recording templates for mice were completed experimentally over time in the lab using blue or black ink pens. The templates were then scanned with an HP Laserjet Pro MFP M525 printer/scanner (Hewlett-Packard, Houston, TX, United States) with standard configuration. In this test, the application was run on a 2.6 GHz Intel Core i5 MacOS 10.13.1 operating system; however, UDReader is compatible with current Macintosh and Windows operating systems. An individual evaluating 672 distinct test tables, using a previously designed Microsoft Excel formula to convert raw data to 50% threshold values resulted in a 96.3% accuracy in 86 min. When the same data was evaluated by UDReader, the accuracy increased to 98.8% with the processing time markedly reduced to 8 min (**Figure 2**).

**FIGURE 3:**
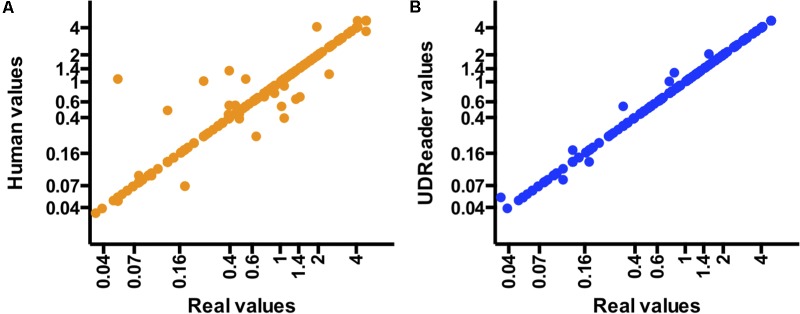
Dispersion of data points highlights the accuracy of UDReader. A comparison between the real values and those obtained by human assessment **(A)** and UDReader **(B)** in the 672 samples evaluated.

**FIGURE 4:**
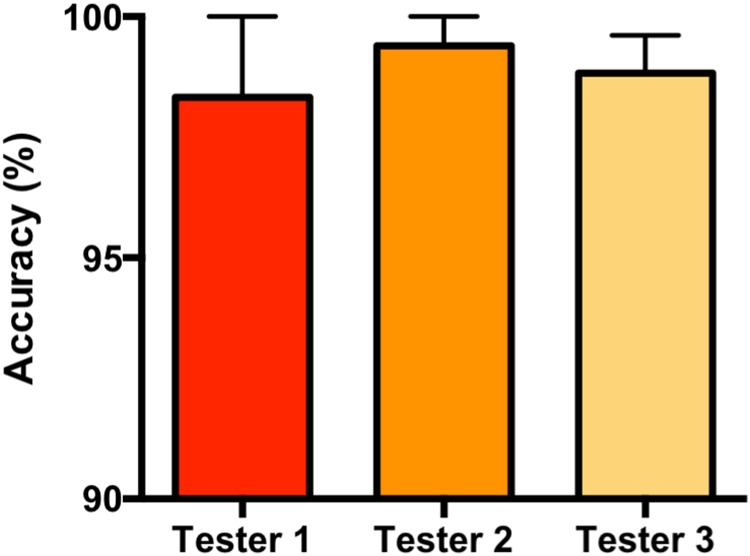
Comparison of UDReader accuracy between the handwriting of three human testers. The 41 tested von Frey sheets were filled out by three human evaluators. The ability of UDReader to correctly calculate thresholds using data from each of these testers was examined. Results demonstrate the average accuracy per sheet per tester, with error bars indicating mean ± SEM.

Furthermore, human errors mainly occurred due to misaligning filament values between tables, brought on by repetitive processing of data across a sheet of repeated tables. This can result in the final recorded 50% value changing significantly (**Figure 3A**). In contrast, the most common errors of UDReader were due to poor identification of a symbol within individual boxes, which result in a 50% value closer to the correct value (**Figure 3B**). Although the set of results analyzed was obtained from various researchers, we wanted to check whether the detection efficiency of UDReader varied depending on the handwriting. We verified that despite the fact that the recognition of the characters is fundamental, the outcome does not show great differences between evaluators (**Figure 4**). If the templates are completed with care and taking into account that the reading by the machine can further reduce the error rate, we propose this as an efficient way of reading primary data tables and calculating 50% von Frey thresholds.

## Conclusion

To simplify the process of calculating 50% threshold values, which previously required searching tabulated result tables or applying cumbersome Microsoft Excel formulae, we have developed the UDReader application. This software reads hand-produced result sheets which are scanned to PDF document and evaluated by a combination of machine vision and machine learning libraries. It is able to quickly and efficiently calculate 50% threshold values from these primary data sources. We demonstrate this application reduces the processing time and errors committed in the process.

UDReader is an open-source program and can be downloaded from https://sourceforge.net/projects/updownreader/ for free. If you find this tool useful in your research studies, referencing this manuscript will be greatly appreciated. The application is free and can be used and modified according to the license provided along with the software. Additional features, improvements, bugs, and general suggestions for future versions of the application can be discussed in the discussion section of our Source Forge page.

## Author Contributions

MC and RG-C: conceptualization. RG-C and BB: software development. MC, RG-C, BB, and DB: formal analysis. RG-C and DB: data curation. MC, RG-C, BB, DB, NA, and LC: writing – original draft; writing – review and editing. MC and RG-C: supervision. MC: project administration. RG-C and MC: funding acquisition.

## Conflict of Interest Statement

The authors declare that the research was conducted in the absence of any commercial or financial relationships that could be construed as a potential conflict of interest. The reviewer AM and handling Editor declared their shared affiliation.

## Footnotes

1 https://opencv.org/releases.html

2 https://www.python.org/downloads/
