# Supplementary material for: Up–Down Reader: An Open Source Program for Efficiently Processing 50% von Frey Thresholds
Source: Front Pharmacol. 2018 May 1;9:433. doi: 10.3389/fphar.2018.00433 (PMC5938897; doi:10.3389/fphar.2018.00433)
Supplement: Supplementary file 1 [file Presentation_1.PDF]

Up-Down Reader

Software User Manual

Table of Contents

1. License 2

2. Software Description 2

3. Installation 2

3.1 Mac OS 2

3.2 Windows 2

4. Usage 3

4.1 PDF input 3

4.2 Image input (JPEG, PNG) 5

5. Support 5

List of Figures

Figure 1: Graphical User Interface Explained 4

Figure 2: On-Screen Output Obtained From a 6-page PDF Input 4

Figure 3: Sample Output File for a 6-page PDF Input 5

1. License

MIT License

Copyright (c) 2017 Bruno Boivin, Rafael Gonzalez Cano

Permission is hereby granted, free of charge, to any person obtaining a copy of this software and associated documentation files (the "Software"), to deal in the Software without restriction, including without limitation the rights to use, copy, modify, merge, publish, distribute, sublicense, and/or sell copies of the Software, and to permit persons to whom the Software is furnished to do so, subject to the following conditions:

The above copyright notice and this permission notice shall be included in all copies or substantial portions of the Software.

THE SOFTWARE IS PROVIDED "AS IS", WITHOUT WARRANTY OF ANY KIND, EXPRESS OR IMPLIED, INCLUDING BUT NOT LIMITED TO THE WARRANTIES OF MERCHANTABILITY, FITNESS FOR A PARTICULAR PURPOSE AND NONINFRINGEMENT. IN NO EVENT SHALL THE AUTHORS OR COPYRIGHT HOLDERS BE LIABLE FOR ANY CLAIM, DAMAGES OR OTHER LIABILITY, WHETHER IN AN ACTION OF CONTRACT, TORT OR OTHERWISE, ARISING FROM, OUT OF OR IN CONNECTION WITH THE SOFTWARE OR THE USE OR OTHER DEALINGS IN THE SOFTWARE.

2. Software Description

The purpose of the software is to automatically read von Frey assay measurements from a scanned document and report the 50% pain threshold based on the up-down method.

3. Installation

3.1 Mac OS

The Mac OS version makes use of the console utility *sips*, a scriptable image processing system that comes with the Mac operating system out of the box. Therefore, the Mac OS version of the software is self-contained, that is the executable application file contains all dependencies it needs to perform its task, with the exception of *sips*, which is already part of your system.

3.2 Windows

The Windows version of the software uses ImageMagick, an open-source software suite, to convert images from one format to another. More specifically, the software requires the *convert*

command prompt utility to perform image conversions. ImageMagick further requires GhostScript to manipulate PDF files; GhostScript is a suite of software whose purpose is to render page description language files (PDF).

To install and launch the Up-Down Reader software, please follow the instructions below:

1. Install GhostScript, specifically the Postscript and PDF interpreter/renderer.
   We recommend using the general public version available at:
   https://www.ghostscript.com/download/gsdnld.html

2. Install ImageMagick.
   The Windows binary release are available at:
   https://www.imagemagick.org/script/download.php
   ** When prompted to select additional extensions to install, ensure that the Legacy Utility *convert* is selected.

3. To launch the software, double click the executable file provided as part of the software.

4. Usage

This section describes how to use the software with an image or PDF document as input. This section further explains how to save the output to a file and where to retrieve such file.

4.1 PDF input

To launch the software, double click the application file.

1. Select the input file by clicking the ***Select file*** button.
2. Select the species for which the data was acquired to ensure the software reports the appropriate pain values.
3. If you wish to save the output to a comma-separated file (csv) once the analysis is completed, click ***Save*** and the output will be saved to the same directory as the selected input file. The exact path will be displayed in a popup window upon clicking ***Save***.
4. Click ***Start*** to start analyzing the input file.

Figure 1: Graphical User Interface Explained

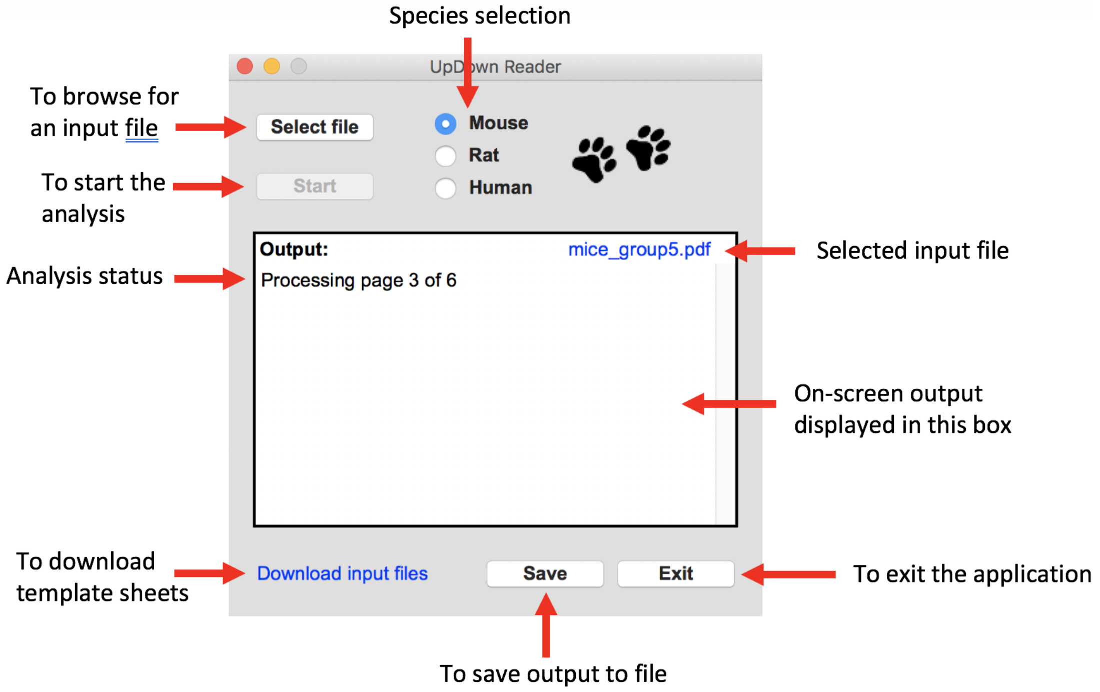

Figure 2: On-Screen Output Obtained From a 6-page PDF Input

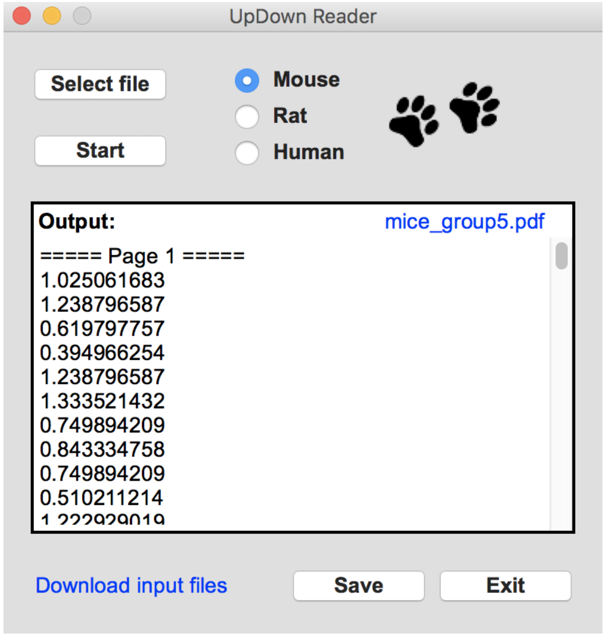

The software is capable of taking as input both single-page and multiple-page PDF files. When provided a multiple-page document, each page gets analyzed independently and is placed in a different column of the output file as shown in figure 3 below.

Figure 3: Sample Output File for a 6-page PDF Input

box_id	page 1	page 2	page 3	page 4	page 5	page 6
1	1.025061683	0.64537181	0.510211214	1.075543638	0.051917048	0.068406605
2	1.238796587	1.075543638	1.025061683	1.075543638	0.090854834	0.12856418
3	0.619797757	0.78398904	0.619797757	0.12856418	0.075962288	0.061668018
4	0.394966254	0.78398904	0.051917048	0.390220419	0.390220419	0.068406605
5	1.238796587	0.440869436	0.350268635	0.619797757	0.474307499	0.075962288
6	1.333521432	0.322894012	0.350268635	0.533408573	0.051917048	0.051917048
7	0.749894209	0.322894012	0.061668018	0.390220419	0.051917048	0.322894012
8	0.843334758	0.322894012	0.78398904	0.090854834	0.0959065	0.440869436
9	0.749894209	1.075543638	1.075543638	1.075543638	0.440869436	0.075962288
10	0.510211214	0.619797757	0.474307499	0.12856418	0.474307499	0.20826677
11	1.222929019	0.350268635	0.350268635	0.12856418	0.474307499	0.12856418
12	1.238796587	1.075543638	0.090854834	0.090854834	0.051917048	0.12856418
13	0.749894209	1.238796587	1.075543638	2.049981775	0.0959065	0.143746752
14	0.749894209	1.075543638	0.390220419	1.027782575	0.394966254	0.322894012
15	0.749894209	0.749894209	0.163632657	0.12856418	0.176135964	0.12856418
16	1.333521432	0.322894012	0.350268635	0.533408573	0.051917048	0.051917048
17	0.749894209	0.322894012	0.061668018	0.390220419	0.051917048	0.322894012
18	0.843334758	0.322894012	0.78398904	0.090854834	0.0959065	0.440869436
19	0.749894209	1.075543638	1.075543638	1.075543638	0.440869436	0.075962288
20	0.510211214	0.619797757	0.474307499	0.12856418	0.474307499	0.20826677

4.2 Image input (JPEG, PNG)

To analyzed individual image files, repeat the procedure outlined above in section 4.1, this time selecting the desired image as input.

5. Support

For additional support, questions, feedback, or to report bugs, please contact us via our sourceforgenet page at https://sourceforge.net/projects/updownreader/.
